# Treatment of Pain in Cancer: Towards Personalised Medicine

**DOI:** 10.3390/cancers10120502

**Published:** 2018-12-10

**Authors:** Marieke H. J. van den Beuken-van Everdingen, Sander M. J. van Kuijk, Daisy J. A. Janssen, Elbert A. J. Joosten

**Affiliations:** 1 Centre of Expertise for Palliative Care, Maastricht University Medical Centre (MUMC+), 6229HX Maastricht, The Netherlands; daisyjanssen@ciro-horn.nl; 2 Department of Clinical Epidemiology and Medical Technology Assessment, Maastricht University Medical Centre (MUMC+), 6229HX Maastricht, The Netherlands; sander.van.kuijk@mumc.nl; 3 Centre of Expertise for Chronic Organ Failure (CIRO), 6085NM Horn, The Netherlands; 4 Department of Anesthesiology and Pain Management, Maastricht University Medical Centre (MUMC+), University Pain Centre Maastricht (UPCM), 6229HX Maastricht, The Netherlands; bert.joosten@mumc.nl; 5 Department of Translational Neuroscience, School of Mental Health and Neuroscience, Maastricht University, 6211LK Maastricht The Netherlands

**Keywords:** cancer pain, prevalence, barriers, undertreatment

## Abstract

Despite increased attention to cancer pain, pain prevalence in patients with cancer has not improved over the last decade and one third of cancer patients on anticancer therapy and half of patients with advanced disease still suffer from moderate to severe pain. In this review, we explore the possible reasons for the ongoing high prevalence of cancer pain and discuss possible future directions for improvement in personalised pain management. Among possible reasons for the lack of improvement are: Barriers for patients to discuss pain with clinicians spontaneously; pain measurement instruments are not routinely used in daily practice; limited knowledge concerning the assessment of undertreatment; changes in patients’ characteristics, including the ageing of the population; lack of significant improvement in the treatment of neuropathic pain; limitations of pharmacological treatment and lack of evidence-based nonpharmacological treatment strategies. In order to improve cancer pain treatment, we recommend: (1) Physicians proactively ask about pain and measure pain using assessment instruments; (2) the development of an optimal tool measuring undertreatment; (3) educational interventions to improve health care workers’ skills in pain management; (4) the development of more effective and personalised pharmacological and nonpharmacological pain treatment.

## 1. Introduction

Despite increased attention to cancer pain, pain prevalence in cancer patients has not significantly changed over the last decade compared to the four decades before. Back in 2007, a systematic review about the prevalence of pain in cancer comprising 40 years (1966–2005), with 52 articles included, and based on almost 20,000 patients, made clear that over 1/3 of patients on anticancer therapy suffered from moderate to severe pain and almost half of the population receiving palliative care had moderate to severe pain [1]. The update of the review on the prevalence in cancer 10 years later showed improvement in attention for pain in cancer patients: A 35 percent increase in papers on the prevalence of pain in cancer were published in the period between 2005 and 2014 compared to the 40 years before. In addition, the methodological quality of these papers increased from only 1 out of 3 being of acceptable quality to over half, and the number of included patients tripled [2]. Unfortunately, pain prevalence did not change and still almost half of the population receiving palliative care experienced moderate to severe pain (Table 1).

Although textbooks often associate malignancies with either a high risk (bone, pancreas, oesophagus) or with a low risk of pain (lymphoma, leukaemia, soft tissue) [4,5], it is not clear what evidence these statements are based on. A cohort study of end-stage cancer patients [6] concluded that cancer pain was not restricted to specific tumour locations. Reyes-Gibby et al. studied patients under anticancer treatment and reported high prevalences of moderate to severe pain in patients with head and neck, gastrointestinal, and breast malignancies [7]. Nevertheless, the type of cancer was not shown to be a predictor of pain prevalence, although patients with gastrointestinal, lung, breast, other haematological, and “other” malignancies had a significantly higher risk of moderate to severe pain than prostate cancer patients [8].

The results of systematic literature reviews raise important questions: Why does pain prevalence not decrease despite inevitably more research on cancer pain? The latter not only resulted in an increased understanding of cancer pain mechanisms, such as pain due to bone metastases [9], but also in the development of new drugs for cancer pain, such as the rapid onset opioids for breakthrough pain. 

Do we still neglect to ask our cancer patients if they experience pain, which as a result might lead to undertreatment? Or might we ask the question if cancer patients and/or their pain has become more complicated over the years or we do not have the tools to treat them properly? In this review, we discuss the possible reasons for the ongoing high prevalence of cancer pain and point at possible future directions for improvement in casu personalised medicine for patients with cancer experiencing pain.

## 2. Pain Prevalence: Reasons for Lack of Improvement in Cancer Pain

### 2.1. Patients do Not Report Pain Spontaneously

A recent study confirmed that cancer patients are still uncomfortable with discussing many of their symptoms in the clinical setting, even when the symptoms are bothersome [10]. Although lack of energy and pain were most often discussed, only about 50% of patients intended to discuss these bothersome symptoms with their physician. Well known patient barriers towards reporting pain, such as misconceptions about analgesic use (fear of adverse effects, addiction, tolerance, and lowered immunity caused by pain medicine), concerns about pain communication (unde-reporting of symptoms to avoid distracting physician from providing cancer treatments), and a belief that pain is inevitable and uncontrollable, are still very prevalent [11]. A study of 135 patients with audio-recorded consultations revealed that patients’ active communication about pain (patients asked questions, stated preferences about pain-related matters, and expressed concerns) made physicians change their pain management regimen, which could lead to better pain control [12]. However, a systematic review of the effectiveness of patient-based educational interventions to improve cancer-related pain concluded that improvement of pain was only seen in less than one third of the studies and in less than 20% of all patients included [13]. The patient-based educational interventions as described (Oldenmenger 2018) varied widely in content and intensity. Furthermore, secondary significant differences were noted in favour of the experimental arms in pain knowledge, relieving barriers, medication adherence, and self-efficacy. From this, it was concluded that standardised patient training programmes addressing clinician–patient communication might possibly lead to better cancer outcomes.

For now, the first step is for the physicians to acknowledge the fact that many patients experience cancer-related pain and proactively ask for pain and pain-related complaints explicitly.

### 2.2. Measurement of Pain

The measurement of pain is an issue in itself and it was shown that only 7 to 43% of physicians use the numeric rating scale (NRS) or visual analogue scale (VAS) in their practice, whereas multidimensional questionnaires for pain assessment are rarely used [11]. Moreover, 30% to 51% of physicians thought that patients exaggerated their pain in order to attract attention [14]. Today, we still do not measure pain routinely, except for research purposes. In a recent study in the Netherlands, in only 0.1% of almost a thousand patients visiting an oncology outpatient clinic was an NRS score of pain intensity recorded [15]. A year later, after introducing the new nationwide cancer pain guidelines in the Netherlands, there was still no mention of pain in the vast majority of patients, and an NRS was found only once in the patient records [16]. A reference to pain in the medical record of patients was found in 50%, 28%, and 21% in respectively academic, large peripheral, and small peripheral hospitals. It is not known why health care professionals are reluctant to consequently measure pain in patients with cancer. Possibly, the lack of knowledge and confidence to administer analgesia effectively [17] and/or underestimating the influence of pain in daily life [18] contribute to poor pain assessment in patients with cancer.

### 2.3. Assessment of Undertreatment

To date, the assessment of undertreatment is not a daily practice. The pain management index (PMI) [19] is a helpful tool to assess undertreatment. First, a pain score and treatment score are calculated: Pain score: Mild = 1 (NRS 0–4), moderate = 2 (NRS 5, 6), severe = 3 (NRS 7–10); treatment score: Non-opioid = 1, weak opioid = 2, strong opioid = 3. Next, the pain score is subtracted from the treatment score. A positive result, i.e., a positive PMI, is said to indicate that the patient is adequately treated, whereas a negative PMI indicates undertreatment. It should, however, be noted that the PMI probably underestimates undertreatment. A patient with severe pain and strong opioids, for example, will not receive a negative score on the PMI (NRS = 0), but this patient is definitely not adequately treated. Furthermore, the sensitivity of PMI scores of < −1 and < 0 for predicting pain interference were only 0.16 and 0.37, and the corresponding specificities were 0.95 and 0.71, respectively. This implies that a negative PMI does not always indicate inadequate pain management [20].

The NRS is likely not the best instrument to assess a patient’s pain. Patients determine whether their pain is ‘controlled’ by whether or not they are able to maintain relationships with family or friends and perform activities which are important to them. The NRS does not necessarily reflect these aspects [21]. Last, but not least, the PMI does not include antineuropathic comedication. For now, however, as the NRS is the best available instrument for the assessment of pain, it is questionable if solid conclusions about the undertreatment of pain in patients with cancer can be made. For future studies and daily practice, the global assessment of the response or pain relief is an easy to use, patient-centered help to assess the adequacy of pain treatment.

### 2.4. Undertreatment

During the last decade, two meta analyses on undertreatment in cancer pain were published. Both concluded that although 30% of patients were still not adequately treated, an improvement over time could be observed [22,23]. The undertreatment of patients was found to be worse in low-income countries and in noncancer hospitals [22,23].

A review of the knowledge and attitudes of healthcare professionals towards cancer-related pain concluded that there is a continuing deficit in professional knowledge regarding cancer pain but also that many of the studies that investigated the effect of educational programmes on healthcare professionals’ knowledge, attitude, and/or practice failed to show an improvement in knowledge, attitude, and/or practice [17]. A discrepancy between self-assessment and actual practice in cancer pain management by medical oncologists, indicating that specialised physicians are not fully aware of their knowledge deficiencies, has been reported [24]. Furthermore, a recent study by Martens et al. noted that with long-term geriatric care, doctors base their choices in opioid prescriptions almost exclusively on personal experience and are barely influenced by guidelines [25]. From this, we conclude that new strategies for teaching healthcare professionals are needed. For instance, improved pain education in undergraduate medical and nursing curricula might help to overcome these problems and introduce guidelines for the treatment of pain in cancer patients at an early stage in teaching and training [26].

### 2.5. Pain and/or Patients More Complex?

#### 2.5.1. Pain Characteristics

Cancer pain can be classified in different ways: By its origin (somatic, neuropathic, or visceral), by its duration (acute or chronic), and by its pattern (continuous and incident or fast and short breakthrough pain).

**Figure 1:**
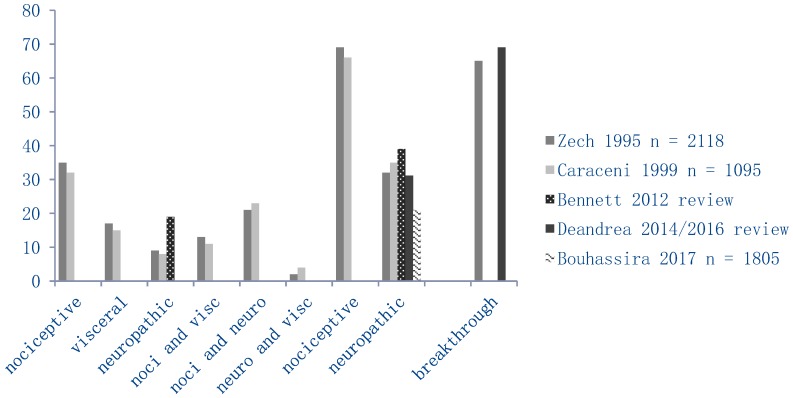
Prevalence (in %) of different pain modalities in cancer, as reported by various studies.

The treatment of cancer pain is more complex when it comes to neuropathic pain and pain with breakthrough. Neuropathic pain, incident pain, and pain intensity are independently associated with the amount of days necessary to achieve stable pain control [27]. Cancer patients with neuropathic pain are most prone to being treated insufficiently. In a recent study among 892 cancer patients, 40% of the patients with moderate to severe pain also showed neuropathic pain symptoms, causing increased interference with daily activities. However, only 8% of these patients were treated with co-analgesics [28]. Patients with neuropathic cancer pain have significantly greater analgesic requirements and have a reduced performance status. These cancer patients with neuropathic pain report worse physical, cognitive, and social function [29]. Patients with breakthrough cancer pain (BTcP) reported significantly higher pain intensity scores than patients without BTcP and they reported an impaired quality of life (QoL) [30]. The prevalence of both BTcP and (partly) neuropathic pain has not changed over the years [31,32,33,34,35,36] (Figure 1).

Over the last 10 years, significant progress has been made in the field of BTcP, both with respect to the definition of BTcP as well as its treatment. Although there are still no universally accepted diagnostic criteria for BTcP, the definition of the task group of the Science Committee of the Association for Palliative Medicine of Great Britain and Ireland [37] is now widely accepted: BTcP is ‘a transient exacerbation of pain that occurs either spontaneously, or in relation to a specific predictable or unpredictable trigger, despite relatively stable and adequately controlled background pain’. The use of this definition allows comparisons of different studies.

The USA already approved oral transmucosal fentanyl citrate (OTFC) for the treatment of BTcP in 1998. Europe followed in 2002 [38]. Since then, many rapid onset opioids (ROOs), all transmucosal fentanyl products, have found their way to the market place. These ROOs follow the pattern of BTcP: Fast and short.

Current BTcP guidelines almost invariably endorse the preferential use of ROOs in breakthrough pain. However, the scientific evidence to support the current guidelines remains at a low grade [39]. 

Much less progress has been made in the treatment of neuropathic pain. It has been established that only a minority of patients with neuropathic pain receive co-analgesics, and that the available adjuvant analgesics have moderate numbers needed to treat (NNT). The pooled NNT of trials with tricyclic antidepressants (TCA), serotonin–noradrenaline reuptake inhibitors (SNRI), and gabanoids are 4–5, 5–7, and 6–12, respectively [40]. 

In conclusion, a modest success of a better treatment of BTcP is observed, and at the same time, a lack of improvement in treatment success in cancer patients with neuropathic pain. These findings cannot fully explain the fact that the overall prevalence of pain in cancer patients did not considerably change over time.

#### 2.5.2. Patient Characteristics

Due to increasing life expectancy and ageing of the population, the prevalence of cancer and cancer treatment in patients over 70 years of age is expected to further increase. Age is associated with and partly influences clinical decisions and outcomes in different ways. Age influences how quickly patients are referred to specialised care. Older patients are often underrepresented in clinical trials, and the number of comorbidities increase and treatment outcomes of the cancer are less favourable [41]. On the other hand, a younger age, in addition to psychological distress and the already mentioned neuropathic pain and incident pain, is independently associated with more days to achieve stable pain control [27]. 

More and more chemotherapy has been prescribed to more and more vulnerable patients over the last 10 years, but whether this has influenced the prevalence of cancer pain is not known. Hence, the present cancer pain polulation significantly differs from the cancer patient population of of a few decennia before. This also implies additional difficulties in the treatment of pain as, for instance, medication is generally not studied in elderly people over 70 years of age, and in this group, comorbidities like heart, lung, liver, and kidney disease, and comedication can cause frequent and/or unexpected side-effects. For example: Clinically important renal impairment is common in old age, and even 99% of patients aged 85 and over have a moderate or severe reduction in the glomerular filtration rate (GFR) [42]. A reduced GFR has significant implications for, amongst others, the choice of strong opioids. The two major metabolites of morphine, morphine-3-glucuronide (M3G) and morphine-6-glucuronide (M6G), are excreted renally [43]. The accumulation of M3G and M6G is already present with mild renal impairment and, in particular, the accumulation of M3G is associated with neurotoxic adverse effects, like hyperalgesia, allodynia, myoclonus, seizures, and delirium [44,45]. Although no recommendations could be formulated on the preferred opioid in patients with renal impairment, based on pharmacokinetics and clinical experience, fentanyl, alfentanil, and sufentanil are mostly recommended [46]. 

It should be stressed that comorbidities can be associated with pain as well. In the elderly, pain is frequent: 65% suffer from osteoarthritic back pain, around 40% of musculoskeletal pain, 35% peripheral neuropathic pain, and 15–25% from chronic joint pain [47].

In conclusion: Although pain characteristics did not change over time, the cancer patients with pain did all the more. The increasing complexity of patients with cancer may at least partly account for the lack of success in reducing pain prevalence in patients with cancer.

### 2.6. Interventions to do Better?

The question remains of, if we just cannot treat cancer pain better than we do now, it is because we just do not have the proper tools to do better. In the last decade, we got access to many different opioids and co analgesics. The disappointing numbers needed to treat for co-analgesics were already mentioned. 

#### 2.6.1. Pharmacological Interventions

**Figure 2:**
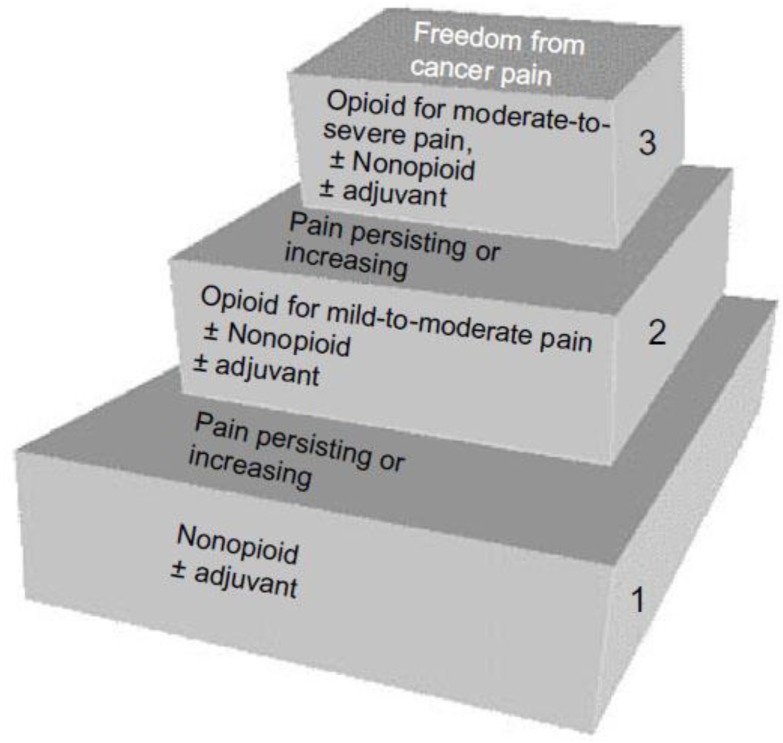
WHO analgesic ladder in cancer pain. Abbreviation: WHO, World Health Organization.

A review of the effectiveness of the use of the WHO pain ladder in cancer pain (Figure 2) showed adequate pain relief in 20–100% of the patients [48]. 

This range in adequate pain relief is quite wide, and depends on the definition of success. When an NRS of <4 is used to define success, 50–90% of the patients can be treated successfully with the use of the WHO pain ladder [32,49].

Although the European Association for Palliative Care proclaimed the WHO ladder essential in treating pain in cancer, they also stated that “there is a shocking lack of evidence to support clinical practice and guidelines at the present time” [50]. Although the WHO ladder is a valuable and easy to follow treatment algorithm to use in the treatment of cancer pain, there is still room for debate. The updated national guideline “treatment of pain in cancer” in the Netherlands recommends prescribing paracetamol as “as needed” medication instead of maintaining the “around the clock” advice with the start of strong opioids (step 3), according to the ladder. Opposed to the positive randomised controlled trial (RTC) of Stocler [51] indicating that adding paracetamol to strong opioids improves pain control and wellbeing, four RCTs [52,53,54,55] and one crossover study [56] failed to demonstrate a beneficial effect of maintaining around-the-clock paracetamol. In a prospective study, 53% of patients wanted to stop taking paracetamol, 18% wanted to continue with regular paracetamol medication as before, and 29% wanted to take paracetamol as needed [57]. A recent Cochrane review concluded: “There is no high-quality evidence to support or refute the use of paracetamol alone or in combination with opioids for the first two steps of the three-step WHO cancer pain ladder” [58].

Another problem with the pharmocological treatment of pain in cancer patients is that numerous studies and meta-analyses up until now have shown no clear benefit in pain relief for one opioid over the other. As a result, there is no consensus on the choice of a strong opioid to start with at step 3 of the WHO ladder [59,60,61,62]. Recent studies tried to make opioid therapy more patient-tailored to improve efficacy by including patient characteristics. For example: Pharmacokinetic studies have shown that drug exposure and/or metabolism significantly differ based on the presence of polymorphisms in genes coding for pharmacokinetics genes. Many potential genetic markers have been described, and the importance of genetic predisposition in opioid efficacy and toxicity has been demonstrated in knockout mouse models and human twin studies. Furthermore, a shortlist of 10 genes that are the most promising markers for clinical has been proposed [63]. The most solid evidence of clinically relevant gene variations on the analgesic treatment with opioids is available for *CYP2D6, COMT, SLC22A1*, and a genetic variant, *OPRM1*. So far, however, the Clinical Pharmacogenetics Implementation Consortium guidelines provide CYP2D6-guided therapeutic recommendations to individualise treatment only with tramadol and codeine. Guidelines for other opioids are lacking [64]. 

As of now, further optimisation of patient/opioid couples is investigated in a different way. Recently, the superiority of methadone over fentanyl in the treatment of neuropathic pain in patients with head-and-neck cancer was shown [65]. These findings are in line with the hypothesis that methadone has a dual mechanism of action, not only interfering with the u-opiate receptor but also with the NMDA-receptor. The latter is known to be specifically involved in neuropathic pain [66]. With this study, the clinical success, defined as a >50% improvement, was significant higher with methadone treatment at 1 week. Demographic characteristics may be important in the prediction of treatment success, as we were able to show that therapy success, again defined as an over 50% pain reduction at one week, was positively associated with the use of methadone, the presence of neuropathic pain, and duration of pain in months [67]. Treatment success was negatively associated with the age of the patient in years. The inclusion of these four characteristics into a prediction model resulted in an area under the curve of almost 82 percent. In line with these findings, Corli and colleagues [68] assessed the role of demographic characteristics, pain features, comorbidities, and ongoing therapy on the lack of efficacy in the treatment of pain in cancer and on the occurrence of severe adverse drug reactions. Liver metastases and the presence of BTpC were found to increase the risk for nonresponse. Conversely, a high basal pain intensity significantly decreased the same risk. 

Apart from the differences between pharmacokinetics and pharmacodynamics in ligand–receptor binding as well as pharmacogenetics, strong opioids differ in the routes of administration and effectiveness. Oral, transdermal, subcutaneous, and intravenous routes of drug delivery are possible for each individual opioid, and this plays an important role in the patients’ choice and wishes. 

In conclusion: There is a need for more effective pain treatments and pain treatment should be personalised using pain and patient characteristics and, in the near future, genetic profiling in order to decrease the prevalence of pain in patients with cancer.

#### 2.6.2. Nonpharmacological Interventions

Already many decades ago, Dame Cicely Saunders introduced the term ‘‘total pain’’ to characterise the multidimensional nature of a patient’s cancer pain experience to include the physical, psychological, social, and spiritual domains. Based on this view and definition, the use of nonpharmacological interventions in addition to pharmacological strategies has been increasing. Nonpharmacological interventions can be divided into two groups: Interventions that aim for patient empowerment and interventons aimed at providing comfort for patients. 

Patients struggle with misconceptions about pain medication, concerns about pain communication, and beliefs about the inevitability and uncontrollability of cancer pain [69]. Many interventions have been developed and evaluated in order to improve pain control and support the self-management of patients. Interventions focused at empowerment of the patient incude addressing knowledge about pain, pain medication, side effects, and alternative methods to control pain. Other interventions target problem-solving skills and communication skills [69]. Unfortunately, none of the interventions dealing with patient empowerment achieved the desired effects on different outcome measures so far, and the questions remain about the optimal format as well as the content as the combination of intervention components [70]. 

In cancer pain, many complementary interventions to provide more comfort for the patient are described: Aromatherapy, with or without reiki and therapeutic touch or massage, massage therapy as such, music therapy, art therapy, and electromyographic biofeedback-assisted relaxation [71]. Unfortunately, most studies are of (very) low quality and show inconsistent results on these same nonpharmacological interventions [71]. Other barriers to applying nonpharmacological interventions are the need to hire external professionals with specific training, and the financial cost for institutions. Properly designated studies are needed to define the place of these interventions in the personalised treatment of cancer pain.

## 3. General Considerations Concerning “Cancer Pain”

Over the last years, the question of whether “cancer pain” is an entity by itself and whether we do not create a “sham distinction” is now known better as a “distinction without a difference“. When pain is experienced by a patient with cancer, this pain is often referred to or indicated as cancer pain. Although this terminology is fairly simple and might suggest easy and straitghforward treatment or treatment algorithms, an indication of “cancer pain” is incomplete and much more complex. For instance, a patient suffering from pain due to bone metastases is familiar with a different type of pain compared to a patient suffering from chemotherapy-induced neuropathic pain. Nevertheless, both types of pain are included within the indication “cancer pain”. Therefore, the indication “cancer pain” should no lomger be used; instead, we should base the indication on the the actual pain syndrome the patient with cancer is suffering from. This might improve the understanding of the underlying pain syndrome, and could assist in the selection of the most optimal drug treatment.

## 4. Conclusions

The prevalence of pain in patients with cancer has remained unchanged over the last decennia despite increased understanding of cancer pain mechanisms and the development of new drugs for cancer pain. One third of cancer patients on anticancer therapy and half of patients with advanced disease still suffer from moderate to severe pain. We discussed various reasons for this lack of improvement: The absence of spontaneuously reporting of pain by patients, insufficient measurement of pain and assessment undertreatment by clinicians, complex pain characteristics, changes in characteristics of patients with cancer, and limitations of interventions, including pain medication. From this, we conclude that the clinician must finally really start measuring pain in cancer patients at all visits and find ways to decrease the “unskilled and unaware” status in health care workers. Currently, educational efforts have limited effects and new and/or earlier interventions have to be sought. Additionally and last, but certainly not least, research into new and more personalised pain treatment must go on, in genetics as well as in pharmacological and nonpharmacological treatment.

## Figures and Tables

**Table 1:** Prevalence of pain in patients with cancer.

	PAIN (%)		NRS >4 (%)	
	1966–2005	2005–2014	1966–2005	2005–2014
after curative treatment	33	39	not reported	27
on anticancer treatment	59	55	36	32
extensive/metastatic disease	64	66	45	52
all stages	53	51	31	33

After curative treatment, 39.3% of patients reported pain; 55.0% during anticancer treatment; and 66.4% in advanced, metastatic, or terminal disease. Moderate to severe pain (numerical rating scale score ≥ 5) [3] was reported by 38.0% of all patients.
